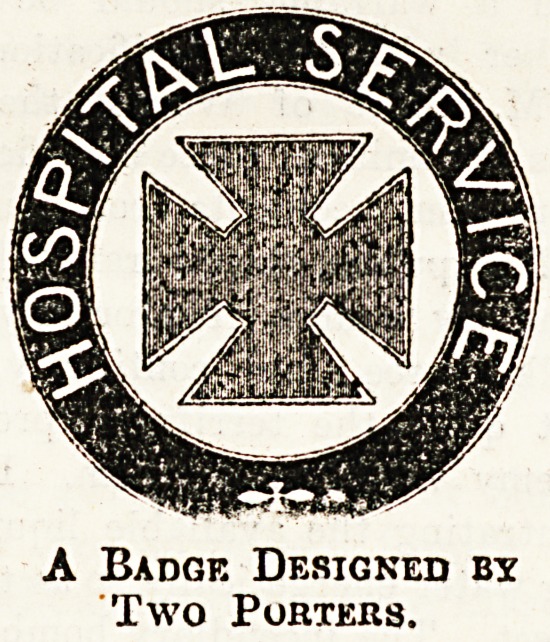# War Service Badges for Hospital Staffs

**Published:** 1915-06-19

**Authors:** 


### WAR SERVICE BADGES FOR HOSPITAL STAFFS.

### What to Avoid and What To Do.

The provision of war service badges for hospital men of military age (or appearance!) is not quite the elementary or minor problem that it appears, and a glance at the letters to be found in our correspondence columns this week is sufficient, apart from other evidence, to show how many individuals and institutional managers are thinking seriously about it. As we noted in our last issue, so far has this anxiety gone that the British Hospitals Association has been approached from institutions in many parts of the country, with the suggestion that it should formulate a common line of action. The significant point in this is not the Association's attitude, which is that local conditions vary so much that central action is not advisable, but rather the fact of appeal to the central body. This is sufficient to show the widespread need for advice and help.

Starting from the common fact that recruiting sergeants, and amateur enthusiasts of either sex, press the claims of military service on all and sundry, and that young men engaged in institutional work (and older men, too, for that matter) are

THE BADGE GIVEN BY THE LONDON HOSPITAL.

A BADGE DESIGNED BY TWO PORTERS.

subjected to ridicule and annoyance on their way to and from their work because they are not in khaki, a cry has arisen for common action and a general remedy. For instance, the idea has been put forward that the War Office should recognise a general badge for hospital workers, on the lines of those which have been granted to special constables and other civilians who, not engaged in Army duties, are yet recognised as performing patriotic and necessary services. It is also suggested that the military authorities in different districts should be approached and asked either to grant or recognise a special badge for hospital men who have been specifically recommended by their responsible hospital officer Now it will be noted that these suggestions have this in common, that they both involve an appeal to the military for aid. This is what makes them so unpractical, since from first to last, and no doubt excusably, the military authorities, depending on voluntary recruits, have been most elaborately particular in refusing to certify anyone or recognise anybody as unfit for foreign service, let alone as usefully occupied at home. In fact, the natural mistake has been made of recruiting without discrimination from all ages, all industries, from the married and unmarried; but with the problems arising from this policy we are not concerned here. The point is that to request any military authority, central or local, to recognise a badge for hospital workers is only to court refusal. What, then, can be done?

The simplest and most practical plan appears to be for the committee of each hospital to grant a badge to its regular officials, indicating that they are employed at the committee's institution. The advantage of this is that the statement represented by the badge is incontestable, and raises no questions which might become awkward for the hospital authorities later on. The object of the badge, let us remember, is to meet the need of young hospital officers who are ridiculed in the streets by laymen or pestered by recruiting sergeants. That is why a badge merely indicating that the wearer is at work at such-and-such a hospital meets the difficulty. If an efficiency badge were issued, the hospital would be taking upon itself the responsibility of saying which of its staff was unfit to join the Army, a question, of course, which must be left to the military authorities alone. Hospital managers cannot be expected to take any such responsibility; all they can properly do is to state a simple fact that such-and-such a person is in their employment; and this, in practice, is proving sufficient to protect their officers from molestation in the street. It is open to every hospital committee to decide on what conditions the badge shall be granted, but the nature of the above badge has the additional merit of making very few conditions necessary. It is not a certificate; it is merely an identification. There is thus no question of it being officially recognised. It serves its purpose, and protects the individual, which is all that it is intended to do.

The second badge here illustrated has an interest all its own. It was designed by two ingenious hospital porters, who procured and paid for it on their own account under the circumstances mentioned in our correspondence columns. Let us then for a moment compare their design with that of the London Hospital in the first illustration. Each bears the Red Cross on its centre in enamel with a white ground. But while the first is surrounded by the words "London Hospital," the second is inscribed "Hospital Service." For the reasons hinted at above we prefer the former; "Hospital Service" is a phrase which is, on the face of it, less specific and, therefore, less satisfactory. The moral, therefore, emerges that such badges should confine themselves to a statement of fact, and, that they may do so most efficiently, they should be used only, if at all, when granted by a hospital committee.

## Figures and Tables

**Figure f1:**
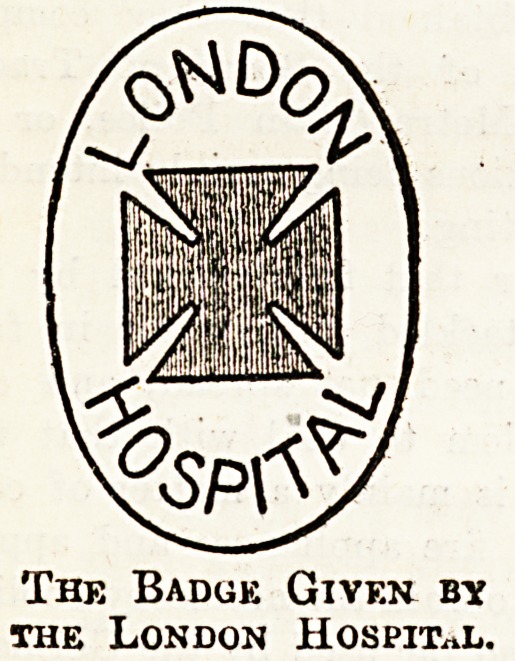


**Figure f2:**